# Invasive group B *Streptococcus* strains and clinical characteristics in Danish infants from 1999 to 2009

**DOI:** 10.3389/fmicb.2022.1001953

**Published:** 2022-09-28

**Authors:** Mads Andersen, Birgitte Smith, May Murra, Stine Yde Nielsen, Hans-Christian Slotved, Tine Brink Henriksen

**Affiliations:** ^1^Department of Clinical Medicine, Aarhus University, Aarhus, Denmark; ^2^Department of Pediatrics and Adolescent Medicine, Aarhus University Hospital, Aarhus, Denmark; ^3^Department of Pediatrics and Adolescent Medicine, Hvidovre Hospital, Hvidovre, Denmark; ^4^Department of Clinical Medicine, Copenhagen University, Copenhagen, Denmark; ^5^Department of Microbiology, Vejle Hospital, Vejle, Denmark; ^6^Department of Biomedicine, Aarhus University, Aarhus, Denmark; ^7^Department of Bacteria, Parasites, and Fungi, Statens Serum Institut, Copenhagen, Denmark

**Keywords:** group B *Streptococcus*, GBS, infection, infants, serotyping, multilocus sequence typing

## Abstract

**Background:**

Group B *Streptococcus* (GBS) infection in infants may result in both respiratory, cardiovascular, and neurological dysfunction and ultimately death of the infant. Surveillance of GBS strains in infants and their clinical characteristics guide development of effective vaccines and other potential treatments and may have implications for future prognostics and infant care. Therefore, we aimed to study GBS serotypes and clonal complexes (CC) in Danish infants with early onset infection (EOD) (0–6 days of life) and late-onset infection (LOD) (7–89 days of life) and to estimate the association between GBS strain and different clinical outcomes.

**Methods:**

We included Danish infants less than 3 months of age with GBS isolates from blood or cerebrospinal fluid between 1999 and 2009. GBS isolates were analyzed by serotyping and multilocus sequence typing with classification of isolates into clonal complexes. Clinical characteristics were obtained by questionnaires completed by tending pediatrician including gestational age, Apgar scores, age at onset, meningitis, symptom severity, treatment duration, and mortality. Symptom severities were reported within neurological symptoms, need for respiratory or circulatory support, and treatment of disseminated intravascular coagulation.

**Results:**

A total of 212 GBS isolates were collected with 129 from EOD and 83 from LOD. The dominating GBS strains were III/CC17 (41%), Ia/CC23 (17%), III/CC19 (15%), Ib/CC8-10 (7%), and V/CC1 (6%). Strain Ia/CC23 was mostly found in EOD, while III/CC17 was widespread in LOD, though being the most common in both EOD and LOD. Strain III/CC17 and Ia/CC23 had highest percentage of samples from cerebrospinal fluid (26%), while III/CC19 had the least (8%). Strain III/CC19 had highest mortality with about one fifth of infected infants dying (22%) followed by Ia/CC23 (16%), Ib/CC8-10 (9%), and then III/CC17 (6%). The symptom severity varied between strains, but with no strain consistently resulting in more severe symptoms.

**Conclusion:**

Some potential differences in disease severity were observed between the different strains. These findings emphasize the continuous need for multimodal surveillance of infant GBS strains and their clinical characteristics to optimize development of GBS vaccines and other potential treatments.

## Introduction

Group B *Streptococcus* (*Streptococcus agalactiae*, GBS) is part of the commensal bacteria in the gastrointestinal tract and vagina and colonizes 10–35% of pregnant women (Khalil et al., 2017; Russell et al., 2017). However, when transmitted to the infant, GBS may result in invasive disease leading to either early onset infection (EOD) defined as an infection within the first 6 days of life or late-onset infection (LOD) defined as an infection between 7 and 89 days of life (Seale et al., 2017). EOD has an estimated global incidence of 205,000 and LOD of 114,000 infants/year (Seale et al., 2017). The introduction of antepartum GBS screening and risk factor-based strategies with intrapartum antibiotic prophylaxis has decreased the incidence of EOD (Lin et al., 2001; Perinatal Group, 2007). GBS screening has decreased the incidence of EOD with about 80% (Perinatal Group, 2007) and is associated with reduced risk compared with risk-based strategies with a relative risk of 0.43 (95% CI: 0.32–0.56) reported in the recent meta-analysis of Hasperhoven et al. (2020). However, these strategies have no effect on LOD and may contribute to development of antibiotic resistance (Lin et al., 2001; Perinatal Group, 2007). Both EOD and LOD may be fatal or cause long-term neurological disabilities (Kohli-Lynch et al., 2017; Seale et al., 2017; Horvath-Puho et al., 2021). The World Health Organization has therefore emphasized the urgent need for GBS vaccination of pregnant women (World Health Organization, 2021). This may reduce the bacterial load in the birth canal and increase transplacental transfer of protective antibodies to the fetus (Madhi et al., 2017). Ten different GBS serotypes have been identified based on GBS capsular polysaccharides (Ia, Ib, II-IX) with serotype III appearing as the most common in both EOD and LOD (Madrid et al., 2017; Bianchi-Jassir et al., 2020). These are currently being investigated as potential vaccine targets with completion of phase III trials now awaiting, covering serotype Ia, Ib, and II-V (Buurman et al., 2019; Carreras-Abad et al., 2020). GBS bacteria may also be classified by different sequence types (ST) by multilocus sequence typing (MLST). MLST examines allelic variations of seven different housekeeping genes (*adhP, atr, glnA, glcK, pheS, sdhA*, and *tkt*), further categorizing similar allelic profiles into clonal complexes (CC) (Jones et al., 2003). The different serotypes and clonal complexes vary by geography and invasiveness, while potential differences in clinical characteristics of the GBS strains have been studied less exhaustively (Jones et al., 2006; Martins et al., 2007; Poyart et al., 2008; Manning et al., 2009; Bekker et al., 2014; Madrid et al., 2017; Kao et al., 2019; Lo et al., 2019; Bianchi-Jassir et al., 2020; Zhu et al., 2020; Baeringsdottir et al., 2021). Surveillance of infant GBS strains and their clinical characteristics may guide development of effective vaccines and other potential treatments. Moreover, it may have implications for future prognostics and infant care. In Denmark, a risk-based approach was implemented in 1997 to identify pregnant women with increased risk of having a newborn developing GBS infection (Khalil et al., 2019). The incidence and serotypes of invasive GBS isolates in Danish infants have previously been reported from 1984 to 2002 and from 2005 to 2018 (Ekelund and Konradsen, 2004; Slotved and Hoffmann, 2020). However, no studies on invasive GBS in Danish infants have investigated the distribution of clonal complexes and the association between GBS strain and infant clinical course. Accordingly, we aimed to investigate GBS serotypes and clonal complexes in Danish infants with EOD and LOD from 1999 to 2009 and to estimate the association between GBS strain and clinical characteristics of the infants including gestational age, Apgar scores, age at onset, meningitis, symptom severity, treatment duration, early morbidities, and mortality.

## Materials and methods

### Study population

We included Danish infants less than 3 months of age with invasive GBS infection from 1999 to 2009.

### Group B *Streptococcus* isolates

Invasive GBS isolates were derived from positive cultures of blood and cerebrospinal fluid. EOD was defined as an isolate causing onset of disease within the first 6 days of life and LOD as an isolate causing onset of disease from 7 to 89 days of life. As part of the national surveillance strategy, all Danish Departments of Clinical Microbiology were encouraged to send GBS isolates to the State Serum Institute (SSI) for further analysis. The identification was described previously by Lambertsen et al. (2010b). All GBS isolates were confirmed by inspection of colony morphology on 5% blood agar plates (SSI Diagnostica, Hillerød, Denmark) and by GBS latex agglutination test (Oxoid A/S, Greve, Denmark). Isolates were stored at −80°C in nutrient beef broth containing 10% glycerol (SSI Diagnostica, Denmark).

### Serotyping

Isolates were serotyped by use of GBS latex agglutination tests and/or by the Lancefield method (capillary precipitation method) (Slotved et al., 2002, 2003). For isolates from 1999 to 2003, the Lancefield method was performed using serotype specific GBS antisera (Ia-IX) (SSI Diagnostica, Denmark) and capsular antigens extracted with both 0.1 and 0.2 N HCl, as described in Slotved et al. (2002). From 2003 to 2009, serotyping was performed using the GBS latex agglutination test (SSI Diagnostica, Denmark), as described in Slotved et al. (2003). In this period, the Lancefield method was only used to confirm isolates with an inconclusive result.

### Multilocus sequence typing

Bacterial lysates as DNA templates were prepared by suspending freshly grown colonies from plates in chelex-solution [1 g chelex 100 resin (Bio-Rad Laboratories, Hercules, USA) in 10 ml 1 × TE-buffer (10 mM TRIS, 1 mM EDTA, pH 8)]. Supernatant was acquired by 10 min of boiling followed by centrifugation for 5 min at 15,000 rpm (Lambertsen et al., 2010a). Sequence types were identified as described by PubMLST (accessed 17 Feb 2022).^1^ PCR for each of the seven genes was performed using a 20 μl PCR mix of 10 μl HotstarTaq Mastermix (Qiagen, Hamburg, Germany), 2 μl DNA-template, and 0.2 μM primer. The PCR program was as follows: 15 min at 95°C, 30 cycles of 15 s at 95°C, 30 s at 55°C, 60 s at 72°C, and 10 min at 72°C. Presence and quality of PCR fragments were tested by gel-electrophoresis on 2% E-gels (Invitrogen, Waltham, USA). Excess primers and nucleotides in the solution containing the PCR fragments were inactivated before sequencing with ExoSap IT^®^ as described by the manufacturer (USB Corporation, Cleveland, USA). Both strands of the PCR fragments were sequenced using 0.2 μM of the relevant primer with BigDye Terminator 3.1 Cycle Sequencing Kit and ABI-prisme 3100 Genetic Analyzer (Applied Biosystems, Foster City, USA) (Lambertsen et al., 2010a). Assignment of sequence type to clonal complex was performed using the goeBURST program (accessed 17 Feb 2022)^2^ (Supplementary material 1). Sequence types that shared at least six of seven allelic variants composed a clonal complex.

### Antibiotic susceptibility testing

All isolates were screened for sensitivity to penicillin using 10 μg oxacillin discs (Oxoid A/S, Denmark) on 5% blood agar plates incubated at 36°C with 5% CO_2_. Until 2004, susceptibility was determined according to the National Committee for Clinical Laboratory Standards (NCCLS) and the Swedish Reference Group for Antibiotics (SRGA) (Ekelund and Konradsen, 2004). From 2005 to 2009, susceptibility was determined according to the European Committee on Antimicrobial Susceptibility Testing (EUCAST) (Lambertsen et al., 2014).

### Patient information

When GBS isolates were received and analyzed by the State Serum Institute, the tending pediatrician was encouraged to complete a questionnaire about the infant and the clinical course of the infection (Supplementary material 2). The following information was collected: maternal risk factors, gestational age at birth, birth weight, Apgar scores at 1 and 5 min, age at disease onset, symptom severity, treatment type and duration, early morbidities, and mortality. Maternal risk factors included GBS colonization in vagina or rectum, GBS bacteriuria during pregnancy, maternal fever (≥38°C) during birth, rupture of membranes >18 h, previous birth of child with GBS-infection, and maternal antibiotics >4 h before birth. Infant symptom severity was reported based on child’s presentation and need for treatment in four categories: respiratory support including nasal continuous positive airway pressure (nCPAP) (mild) and mechanical ventilation (severe); circulatory support including fluid-resuscitation (mild) and vasopressor treatment (severe); neurological symptoms including irritability (mild) and seizures (severe); and treatment of coagulopathy including infusion of platelets or fresh frozen plasma (mild) and antithrombin III or cryoprecipitate (severe).

### Statistical analyses

Maternal and infant characteristics were analyzed by EOD and LOD. Incidence of invasive GBS infection was estimated from 1999 to 2009 as number of isolates/1,000 livebirths with lowest and highest yearly incidence during the study period (range). Incidence was calculated based on the assumption that the State Serum Institute received 58% of all GBS isolates in Denmark during the study period as previously described (Ekelund et al., 2005). The annual number of livebirths was obtained from Statistics Denmark (StatBank Denmark, 1850). Serotypes, sequence types, and clonal complexes were studied individually and by pairing these characteristics. We then investigated the association between GBS strain and infant clinical characteristics including gestational age, Apgar scores at 1 and 5 min, age at onset, occurrence of meningitis, symptom severity, treatment duration, early morbidities, and mortality. Only the five most common combinations of serotype and clonal complex were included in these statistical analyses (III/CC17, Ia/CC23, III/CC19, Ib/CC8-10, and V/CC1) with reports of the distribution-percentage within each of these groups. Missing answers were not included in the primary analysis (Supplementary material 3). However, the associations between GBS strain, symptom severity, and mortality were also investigated with sensitivity analyses with all missing values in returned questionnaires recoded as the mildest disease presentation. Continuous and ordinal data were analyzed by Mann Whitney or Kruskal Wallis tests, while categorical data were analyzed by Fisher’s exact tests. All analyses were conducted in Stata 17 (StataCorp. 2021. *Stata Statistical Software: Release 17*. College Station, TX: StataCorp LLC) and GraphPad Prism (Version 8 Mac, GraphPad Software, San Diego, California USA).

### Ethical approvals

This study was approved by the Scientific-Ethical Committees for Copenhagen and Frederiksberg [no. (KF) 01-153/00].

## Results

### Population

A total of 212 infants less than 3 months of age had GBS isolates from blood or cerebrospinal fluid send to the State Serum Institute during the 11-year study period. A total of 171 (81%) clinical questionnaires were returned, but completeness of each varied (Supplementary material 3). The infants were admitted to 17 different Pediatric Departments around the country. Questionnaires were returned from each of the five Danish regions, represented with at least six infants (range: 6–72) (Supplementary material 4). Maternal and infant characteristics are provided in Table 1. EOD occurred in 129 (61%) infants and LOD in 83 (39%). A total of 169 (80%) isolates were sampled from blood, while 43 (20%) were sampled from the cerebrospinal fluid. Prolonged rupture of membranes was more common in EOD than in LOD (25% vs. 10%), while meningitis was more common in LOD (29% vs. 15%).

**TABLE 1:** Information on 212 infants with Group B *Streptococcus* (GBS) isolates by early onset GBS disease (EOD) and late-onset GBS disease (LOD) in Denmark from 1999 to 2009.

	EOD (*n* = 129)^a^	LOD (*n* = 83)^a^
**Infant characteristics**		
Female	56 (43%)	41 (49%)
Preterm	39 (38%)	20 (30%)
Birth weight (g)	3,270 (2,475–3,750)	3,390 (2,430–4,060)
Age at disease onset (days)	1 (0–1)	28 (16–45)
**Maternal risk factors**		
GBS in vagina or rectum	4 (4%)	2 (3%)
GBS bacteriuria	3 (3%)	3 (4%)
Maternal fever (>38°C)	11 (11%)	5 (7%)
PROM (>18 h)	23 (22%)	6 (9%)
Previous child with GBS	0 (0%)	1 (1%)
Antibiotics > 4 h before birth	14 (13%)	4 (6%)
**Clinical**		
Meningitis	19 (15%)	24 (29%)
Treatment duration (days)	11 (8–16)	10 (7–15)
Mortality	11 (11%)	8 (12%)
**Respiratory support^b^**		
nCPAP	47 (45%)	20 (30%)
Mechanical ventilation	15 (14%)	16 (24%)
**Circulatory support^b^**		
Fluid-resuscitation (albumin)	13 (13%)	2 (3%)
Vasopressor	11 (11%)	9 (13%)
**Neurological symptoms^b^**		
Irritabile	47 (45%)	23 (34%)
Seizures	11 (11%)	15 (22%)
**DIC^b^**		
Thrombocytes/FFP	7 (7%)	6 (9%)
AT3/cryoprecipitate	4 (4%)	1 (1%)

Continuous variables were presented as medians with interquartile ranges, while categorical variables were presented as frequencies with percentages. EOD was defined as samples drawn within 6 days of life and LOD between 7 and 89 days of life. PROM, prolonged rupture of membranes; nCPAP, nasal continuous positive airway pressure; DIC, disseminated intravascular coagulopathy; FFP, fresh frozen plasma; AT3, antithrombin 3.

^a^The percentages were based on 171 returned questionnaires (EOD = 104, LOD = 67) with exception of sex and meningitis where information was available for all infants.

^b^An infant could only be assigned to one of the severities (most severe degree).

### Epidemiology

The estimated incidence of GBS infection in Denmark from 1999 to 2009 was 0.5/1,000 livebirths (range: 0.3–0.7/1,000). EOD occurred in 0.3/1,000 livebirths (range: 0.1–0.5/1,000), while LOD occurred in 0.2/1,000 livebirths (range: 0.1–0.3/1,000). The incidence during the study period fluctuated but with no obvious trend. All isolates were fully susceptible to penicillin.

### Prevalences of group B *Streptococcus* strains

**FIGURE 1:**
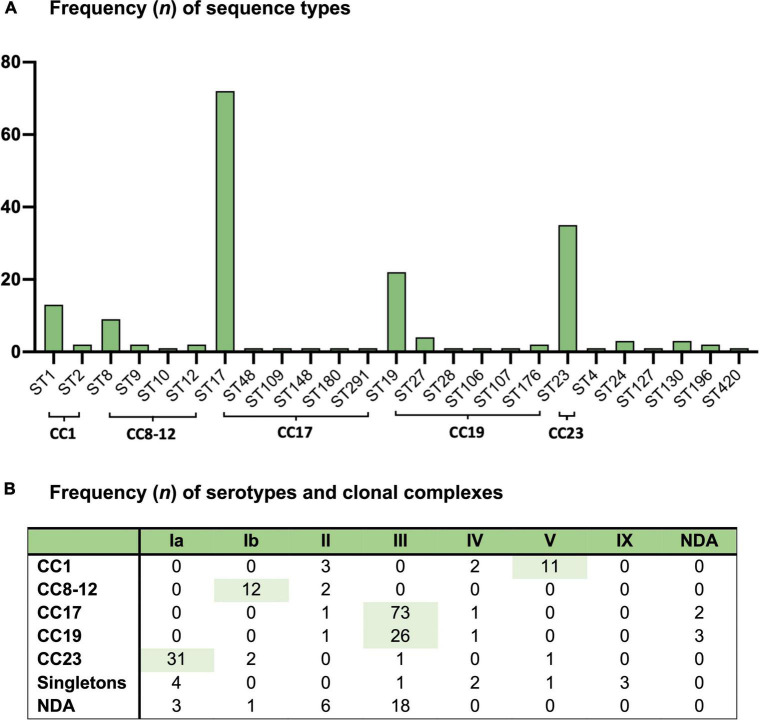
Group B *Streptococcus* isolates from 212 Danish infants within 3 months of life from 1999 to 2009 and **(A)** the frequency of sequence types (ST) and **(B)** the frequency of serotypes (Ia-IX) and clonal complexes (CC). NDA, no data available.

A total of 5 GBS isolates were non-typeable, while we were unable to profile 28 isolates by MLST. The most common serotype was type III (57%) followed by type Ia (18%), type Ib (7%), type II (6%), type V (6%), type IV (3%), and type IX (1%) (Figure 1) (Supplementary material 5). Serotype VI, VII, and VIII were not observed. The most common sequence types were ST17 (39%), ST23 (19%), ST19 (12%), ST1 (7%), and ST8 (5%); with the most common clonal complexes being CC17 (42%), CC23 (19%), CC19 (17%), CC1 (9%), and CC8-12 (8%) (Figure 1) (Supplementary material 5). When looking at the pairing of serotypes with allelic variations; serotype Ia was mostly associated with CC23, type Ib with CC8-10, type III with CC17 and CC19, type V with CC1, and type IX with ST130 (Figure 1). Thus, the most common GBS strains were III/CC17 (41%), Ia/CC23 (17%), III/CC19 (15%), Ib/CC8-10 (7%), and V/CC1 (6%). We observed no statistically significant changes in the prevalence of any GBS strain during the study period (Supplementary material 6).

### Age at onset and meningitis

Evidence of an association was found between GBS strain and age at disease onset with Ia/CC23 being associated with EOD and III/CC17 with LOD, though with III/CC17 being the most common strain in both EOD and LOD (Table 2). Strain Ia/CC23 and V/CC1 had the highest percentage of isolates leading to EOD (25/31 and 9/11, 80%) followed by Ib/CC8-10 (8/12, 67%), III/CC19 (14/26, 54%), and then III/CC17 (35/73, 48%) (Table 2). When looking at the occurrence of meningitis, strains Ia/CC23 and III/CC17 had the highest percentage of isolates from the cerebrospinal fluid (8/31 and 19/73, 26%) followed by Ib/CC8-10 (2/12, 17%), V/CC1 (1/11, 9%), and then III/CC19 (2/26, 8%) (Table 2).

**TABLE 2:** Invasive Group B *Streptococcus* (GBS) strains from Danish infants within 3 months of life between 1999 and 2009 and their clinical characteristics.

	Ia/CC23 (*n* = 31)	Ib/CC8-10 (*n* = 12)	III/CC17 (*n* = 73)	III/CC19 (*n* = 26)	V/CC1 (*n* = 11)	*p*-value
**Clinical**						
Early onset disease	25 (81%)	8 (67%)	35 (48%)	14 (54%)	9 (82%)	0.01
Meningitis	8 (26%)	2 (17%)	19 (26%)	2 (8%)	1 (9%)	0.26
Mortality	4 (16%)	1 (9%)	3 (6%)	5 (22%)	0 (0%)	0.16
**Respiratory support^a^**						
nCPAP	10 (42%)	6 (60%)	19 (39%)	11 (50%)	5 (71%)	0.92
Mechanical ventilation	5 (23%)	2 (20%)	13 (27%)	5 (23%)	0 (0%)	
**Circulatory support^a^**						
Fluid-resuscitation (albumin)	2 (9%)	2 (20%)	1 (2%)	5 (24%)	1 (14%)	0.58
Vasopressor	2 (9%)	2 (20%)	9 (19%)	3 (14%)	1 (14%)	
**Neurological symptoms^a^**						
Irritabile	8 (38%)	4 (40%)	21 (43%)	11 (52%)	6 (67%)	0.27
Seizures	2 (10%)	0 (0%)	10 (20%)	3 (14%)	1 (11%)	
**DIC^a^**						
Thrombocytes/FFP	2 (9%)	1 (11%)	4 (9%)	3 (16%)	1 (14%)	0.99
AT3/cryoprecipitate	1 (4%)	0 (0%)	2 (4%)	0 (0%)	0 (0%)	

The percentages were based on non-missing answers. Data were analyzed by Fisher’s Exact test (categorical variables) and by Kruskal Wallis test (ordinal variables). nCPAP, nasal continuous positive airway pressure; DIC, disseminated intravascular coagulopathy; FFP, fresh frozen plasma; AT3, antithrombin 3.

^a^An infant could only be assigned to one of the severities (most severe degree).

### Other clinical characteristics

No evidence of an association was found between GBS strain and gestational age (Supplementary material 7). The majority of children with EOD had 1 and 5-min Apgar scores of 10–7 (71 and 84%). However, several children also presented with 1 and 5-min Apgar scores of 3–0 (16 and 15%). No apparent difference was observed in Apgar scores between the GBS strains (Supplementary material 7). Most infants (64%) needed respiratory support regardless of the time of onset, with nCPAP being more common in EOD compared with LOD and mechanical ventilation being more common in LOD compared with EOD. Just as many infants (64%) showed symptoms related to the central nervous system with seizures being more than twice as prevalent in infants with LOD. About one fourth of the infants (24%) needed circulatory support including fluid-resuscitation or vasopressors, while about one eighth (13%) were treated for disseminated intravascular coagulation (Table 1). The symptom severity within these categories varied somewhat by strain. However, no strain consistently resulted in more severe symptoms (Table 2). A total of 19 infants died (11%) (Table 1). Strain III/CC19 was associated with the highest mortality with death occurring in about one out of five infants infected by that strain (5/23, 22%) followed by Ia/CC23 (4/25, 16%), Ib/CC8-10 (1/11, 9%), and then III/CC17 (3/54, 6%). The most common antibiotics used were ampicillin (86%) and penicillin (83%), often in combination with gentamicin (67%). The median treatment duration among all surviving infants was 11 days (IQR: 8–15 days) (Table 1). The median treatment duration was highest for III/CC19 with 15 days (IQR: 10–22 days) (Supplementary material 8).

### Early morbidities

Early morbidities related to the GBS infections included one brain abscess (III/CC17), one neck abscess (Ib/CC8-10), one with cerebral and pulmonary hemorrhage (Ia/CC23), and two with hypoxic-ischemic brain lesions (III/CC17). In addition, 11 infections were complicated by osteomyelitis or septic arthritis (III/CC17 = 4, III/CC19 = 3, Ib/CC8-10 = 2, V/CC1 = 1, V/ST127 = 1).

### Sensitivity analyses

Sensitivity analyses with recoding of missing answers to mildest disease presentation failed to change the association between GBS strain and symptom severity and mortality (Supplementary material 9).

## Discussion

We examined 212 invasive GBS isolates from Danish infants during an 11-year period with about 60% resulting in EOD and 40% resulting in LOD. Serotype III was present in more than 50% of the infants, and type Ia in approximately 20%. A hexavalent vaccine including serotype Ia, Ib, and II-V would have covered 95% of all typeable strains in this study (Buurman et al., 2019; Carreras-Abad et al., 2020). Slotved and Hoffmann (2020) described GBS serotypes in Danish infants from 2005 to 2018 and found similar prevalences as the current study, indicating stable serotype distribution over several decades with unchanged use of intrapartum antibiotic in high risk women. The most common GBS strain was III/CC17 followed by Ia/CC23, III/CC19, Ib/CC8-10, and then V/CC1. This distribution of serotypes and clonal complexes correspond to that reported in the meta-analysis by Bianchi-Jassir et al. (2020). In Europe and North America, serotype III was the most common in infant invasive disease (67%) followed by type Ia (17%), V (7%), Ib (4%), and II (4%). This was somewhat comparable to other continents with the largest relative difference in serotype distribution being Ib accounting for 12% in Asian countries. We found type Ib to account for 7% of all serotypes among Danish infants. With regards to clonal complexes, they similarly found CC17 to be the most common clonal complex (42%) followed by CC19 (18%) and then CC23 (15%). We found that CC23 was the second most common clonal complex accounting for 20%, while CC19 accounted for 18%. Among the GBS strains, we found that Ia/CC23 particularly caused EOD, while III/CC17 was widespread in LOD, although being the most common strain in both EOD and LOD. This is also in accordance with previous findings from the meta-analysis by Bianchi-Jassir et al. (2020). Strains of CC17 have been reported to be more virulent compared with other clonal complexes, which may be due to the expression of the CC17-specific hypervirulent GBS adhesin (HvgA) (Jones et al., 2006; Martins et al., 2007; Tazi et al., 2010). This may challenge future vaccine development as capsular switching within this lineage has been observed and hypervirulence may be independent of the serotype (Jones et al., 2006; Bellais et al., 2012; Khan et al., 2022).

Similar to previous reports, we found that the prevalence of meningitis was higher in LOD than in EOD (Bianchi-Jassir et al., 2020). Strain III/CC17 had among the highest percentage of isolates causing meningitis, while III/CC19 had the least, though with no apparent differences in neurological symptoms. Strains of CC17 have previously been associated with the development of meningitis (Poyart et al., 2008; Manning et al., 2009), potentially due to an enhanced ability of CC17 to pass the blood-brain barrier through expression of specific membrane adhesins (Srr2) interacting with host integrins (Lentini et al., 2018; Deshayes de Cambronne et al., 2021). Despite this association, we found that strain III/CC17 had among the lowest mortalities between the different GBS strains, while III/CC19 showed the highest. This may indicate that even though CC17 is highly virulent, it may result in less severe disease compared with other strains. We also found that the need for circulatory support was almost twice as high in infants infected with strain III/CC19 compared with III/CC17, which was solely explained by more infants needing fluid-resuscitation. Furthermore, the median duration of antibiotic treatment was about 4 days longer in strains of III/CC19 compared with III/CC17. The increased duration of antibiotic treatment in infants with strain III/CC19 could be an accidental finding or due to the expression of certain resistance genes within this lineage including the *pbp2x* gene or macrolide-lincosamide-streptogramin resistance genes (Metcalf et al., 2017; Jamrozy et al., 2020). Though, higher resistance-patterns in III/CC17 has recently been reported in other populations (Kao et al., 2019; Plainvert et al., 2020). The differences in disease severity between III/CC17 and III/CC19 indicate that the clinical outcomes related to serotype III may be highly influenced by the associated clonal complex. As we found that these two strains had an almost similar distribution of isolates causing EOD and LOD, age of onset could not explain these differences in disease severity. CC19 is the most common clonal complex in infant colonization accounting for 40% of all strains, while CC17 only accounts for 5% (Bianchi-Jassir et al., 2020). Instead, it may be that CC19 more often infect children with compromised immune systems such as preterm newborns, which in itself may be associated with higher mortality. Despite finding no overall association between GBS strains and gestational age, 45% of infants infected with strain III/CC19 and 32% with strain III/CC17 were born prematurely. However, differences in genetic and molecular characteristics could still be involved (Shabayek and Spellerberg, 2018; Flaherty et al., 2019). Serotype III has previously been associated with lower mortality and fewer neurological complications including seizures compared with other serotypes such as type Ia, type Ib, and type V (Kao et al., 2019; Lo et al., 2019; Zhu et al., 2020). Our finding of different clinical courses between III/CC17 and III/CC19 highlights the importance for future studies to include both serotypes and clonal complexes, when investigating the association between GBS strain and clinical characteristics. Previous studies have found that serotype Ib and CC10 were associated with respiratory difficulties; while one study has reported that serotypes Ib and V were associated with higher rates of disseminated intravascular coagulation (Kao et al., 2019; Zhu et al., 2020; Baeringsdottir et al., 2021). We were unable to substantiate these findings in our population due to small numbers of these strains and few events of disseminated intravascular coagulation.

This is the first Danish study on infants to report the prevalence of both GBS serotypes and clonal complexes and to investigate the association between strains and their clinical characteristics. With inclusion of 212 isolates, this study is also among the largest investigations in general of the clinical course of the infection by strain (Bianchi-Jassir et al., 2020). However, some limitations need to be considered. Despite the large number of isolates, some strains and clinical events were still rare, which thus led to imprecision of the estimates. It has previously been estimated that the State Serum Institute received around 58% of all GBS isolates in Denmark around the study period (Ekelund et al., 2005). However, as isolates were received from all Danish Pediatric Departments, we believe to have obtained a representative sample. As the questionnaires were completed by the tending pediatrician, several replies on clinical outcomes were also missing. However, as we expect missing values to be independent of the specific GBS strain, we also believe it unlikely that this would have biased the association between strain and clinical course. In addition, results from sensitivity analyses were essentially unchanged from the complete-case analyses. Though, the pediatricians may overall have been more inclined to report more severe cases as these may generate larger impacts. We only reported on penicillin resistance, as information on resistance and susceptibility to other antibiotics was unavailable on an individual level. However, the frequency of antibiotic resistance and susceptibility to both penicillin, clindamycin, and erythromycin among all GBS isolates in Denmark are published in yearly reports from the Danish Integrated Antimicrobial Resistance Monitoring and Research Programme (accessed 10 Aug 2022).^3^ We did not investigate the expression of other GBS surface proteins including different adhesins (Bianchi-Jassir et al., 2020). These proteins should be considered for inclusion in future studies investigating possible vaccine targets and could have provided additional information on how some strains may affect clinical outcomes (Shabayek and Spellerberg, 2018). At last, this study only included infants from 1999 to 2009 and therefore does not contain current trends. However, our GBS serotype distribution was similar to the findings in the previous Danish publication from 2005 to 2018 (Slotved and Hoffmann, 2020).

## Conclusion

Strain III/CC17 was responsible for most GBS infections in Danish infants followed by Ia/CC23, Ib/CC8-10, III/CC19, and V/CC1. Strain Ia/CC23 was associated with EOD and III/CC17 with LOD. Some potential differences in other clinical characteristics were observed between the GBS strains, especially between strain III/CC17 and III/C19 with development of meningitis and mortality appearing to differ. These findings emphasize the continuous need for multimodal surveillance of infant GBS strains and the clinical characteristics of both serotypes and clonal complexes to optimize development of GBS vaccines and other potential treatments.

## Data availability statement

The original contributions presented in this study are included in the article/Supplementary material, further inquiries can be directed to the corresponding author.

## Ethics statement

The studies involving human participants were reviewed and approved by the Scientific-Ethical Committees for Copenhagen and Frederiksberg. Written informed consent from the participants’ legal guardian/next of kin was not required to participate in this study in accordance with the national legislation and the institutional requirements.

## Author contributions

H-CS and BS contributed to the conception, design of the study, and the acquisition of data. MA drafted the manuscript. All authors critically revised the manuscript for important intellectual content, approved the final version for publication, agreed to be accountable for all aspects of the work, and contributed to the analyses, and interpretation of the data for the study.

## Footnotes

1
www.pubmlst.org/sagalactiae

2
www.phyloviz.net/goeburst

3
www.danmap.org/reports

## Abbreviations

AT3: antithrombin 3
CC: clonal complex
DIC: disseminated intravascular coagulopathy
EOD: early onset GBS infection
FFP: fresh frozen plasma
GBS: Group B *Streptococcus*
IQR: interquartile range
LOD: late-onset GBS infection
MLST: multi locus sequence typing
nCPAP: nasal continuous positive airway pressure
NDA: no data available
PROM: prolonged rupture of membranes
ST: sequence type
SSI: State Serum Institute.
