# Supplementary material for: Invasive group B Streptococcus strains and clinical characteristics in Danish infants from 1999 to 2009
Source: Front Microbiol. 2022 Sep 28;13:1001953. doi: 10.3389/fmicb.2022.1001953 (PMC9554412; doi:10.3389/fmicb.2022.1001953)
Supplement: Supplementary file 1 [file Data_Sheet_1.docx]

Supplementary Material

**Title**

Invasive Group B *Streptococcus* strains and clinical characteristics in Danish infants from 1999 to 2009

**Authors**

Mads Andersen^1,2*^, Birgitte Smith^3,4^, May Murra^5^, Stine Yde Nielsen^6^, Hans-Christian Slotved^7^, Tine Brink Henriksen^1,2^

**Affiliations**

^1^Department of Clinical Medicine, Aarhus University, Aarhus, Denmark

^2^Department of Paediatrics and Adolescent Medicine, Aarhus University Hospital, Aarhus, Denmark

^3^Department of Pediatrics, Hvidovre Hospital, Hvidovre, Denmark

^4^Department of Clinical Medicine, Copenhagen University, Denmark

^5^Department of Microbiology, Vejle Hospital, Vejle, Denmark

^6^Department of Biomedicine, Aarhus University, Aarhus, Denmark

^7^Department of Bacteria, Parasites, and Fungi, Statens Serum Institut, Copenhagen, Denmark

Table of Contents

[Supplementary 1. Allelic similarities 3](#_Toc112320342)

[Supplementary 2. English translation of the questionnaire 4](#_Toc112320343)

[Supplementary 3. Missing answers in questionnaires 5](#_Toc112320344)

[Supplementary 5. Distribution of serotypes and clonal complexes in EOD and LOD 7](#_Toc112320345)

[Supplementary 6. Distribution of GBS strains by time-period 8](#_Toc112320346)

[Supplementary 7. GBS strains and other clinical characteristics 9](#_Toc112320347)

[Supplementary 8. GBS strains and treatment duration 10](#_Toc112320348)

[Supplementary 9. Sensitivity analyses 11](#_Toc112320349)

**Abbreviations**

GBS, Group B *Streptococcus* (*Streptococcus agalactiae*)

EOD, early-onset GBS disease (0-6 days of life)

LOD, late-onset GBS disease (7-89 days of life)

AT3, antithrombin III

nCPAP, nasal continuous positive airway pressure

CNS, central nervous system

CPR, personal identification number

DIC, disseminated intravascular coagulopathy

NO/ECMO, nitrogen oxide/extracorporeal membrane oxygenation

# Supplementary 1. Allelic similarities

Overview of the results from the goeBURST program ([www.phyloviz.net/goeburst](http://www.phyloviz.net/goeburst), accessed 24 July 2022) showing allelic similarities between the different sequence types.

**
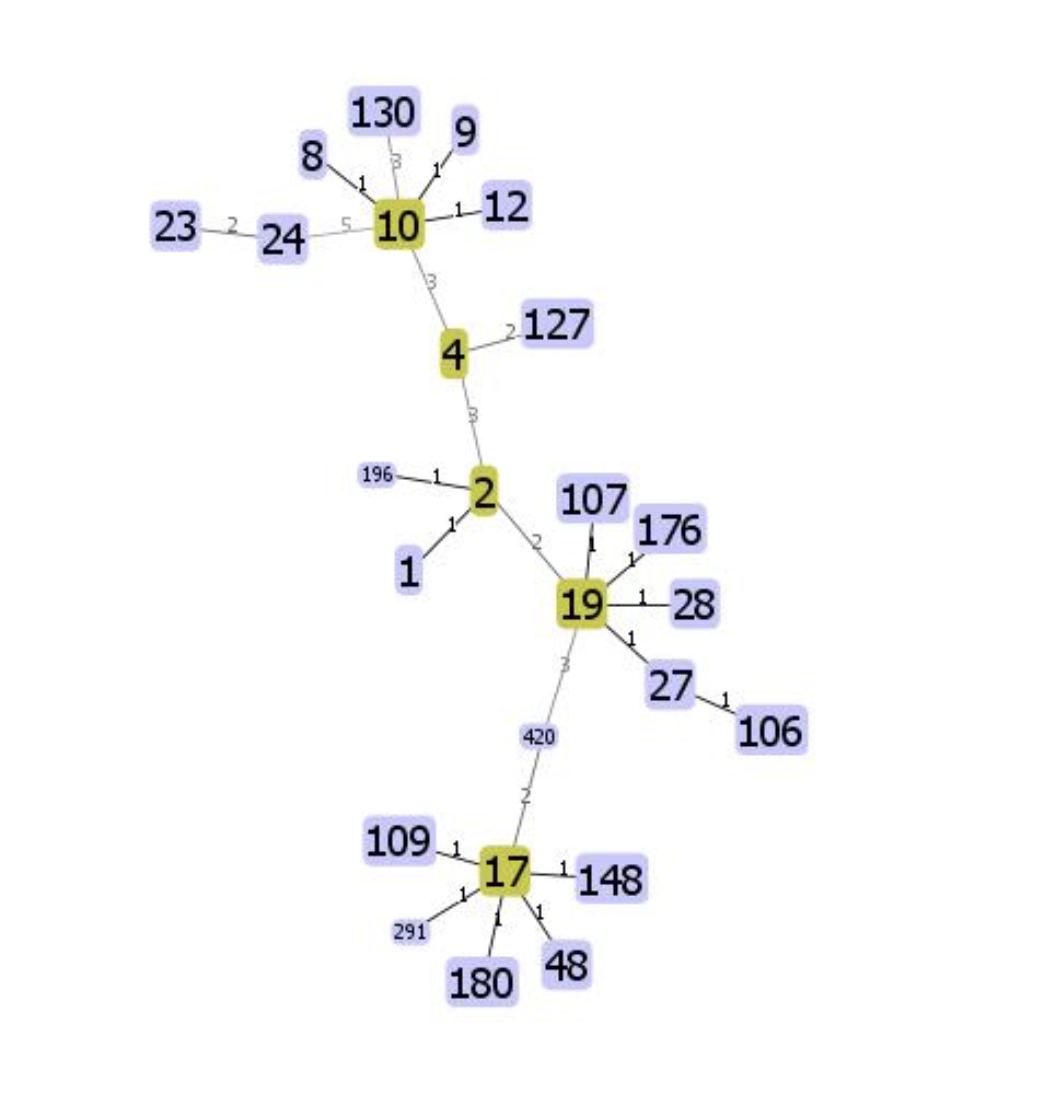
**

# Supplementary 2. English translation of the questionnaire

| **Name (maternal):** | | | | | | | | **CPR (maternal):** | | | | | | | |
| --- | --- | --- | --- | --- | --- | --- | --- | --- | --- | --- | --- | --- | --- | --- | --- |
| **Place of birth:** | | | | | | | | **CPR (child):** | | | | | | | |
| **Place(s) of treatment:** | | | | | | | | | | | | | | | |
|  | | | | | | | | | | | | | | | |
| **Sample type:^1^** | - Blood | | | | | | | Date: | | | | | Test number: | | |
|  | - Cerebrospinal fluid | | | | | | | Date: | | | | | Test number: | | |
|  | | | | | | | | | | | | | | | |
| **Risk factors** | | | | | | | | | | | | | | | |
| Previous (still)birth of child with GBS-infection | | | | | | | | - Yes | | | - No | | | | - Not registered |
| GBS-bacteriuria in concerned pregnancy | | | | | | | | - Yes | | | - No | | | | - Not registered |
| GBS in vagina/rectum in concerned pregnancy | | | | | | | | - Yes | | | - No | | | | - Not registered |
| Maternal fever (≥38 °C) during birth | | | | | | | | - Yes | | | - No | | | | - Not registered |
| Prolonged rupture of membranes >18 hours | | | | | | | | - Yes | | | - No | | | | - Not registered |
| ≥ 2 doses of antibiotics to the mother during birth | | | | | | | | - Yes | | | - No | | | | - Not registered |
| Treatment >4 hours before birth | | | | | | | | - Yes | | | - No | | | | - Not registered |
| Type of antibiotic: | | | | | | | | | | | Number of doses: | | | | |
| Type of antibiotic: | | | | | | | | | | | Number of doses: | | | | |
| Type of antibiotic | | | | | | | | | | | Number of doses: | | | | |
|  | | | | | | | | | | | | | | | |
| Gestation (week and days): | | | | | Birth weight: | | | | | Apgar score (1): | | | | | Apgar score (5): |
|  | | | | | | | | | | | | | | | |
| **Disease severity** | | | | | | | | | | | | | | | |
|  | None | | Mild | | | | | Moderate | | | | | | Severe | |
| Respiratory |  | | - nCPAP | | | | | - Respirator | | | | | | - NO/ECMO | |
| Circulatory |  | | - Albumin | | | | | - 1 vasopressor | | | | | | - ≥2 vasopressors | |
| CNS |  | | - Irritable | | | | | - Seizures | | | | | | - Convulsant | |
| DIC |  | | - Thrombocytes/FFP | | | | | - AT3/cryoprecipitate | | | | | | - Uncontrollable | |
|  | | | | | | | | | | | | | | | |
| **Antibiotics during the course** | | | | | | | | | | | | | | | |
|  | Type | | | | | | Start date | | | | | | | End date | |
| 1 |  | | | | | |  | | | | | | |  | |
| 2 |  | | | | | |  | | | | | | |  | |
| 3 |  | | | | | |  | | | | | | |  | |
| 4 |  | | | | | |  | | | | | | |  | |
|  | | | | | | | | | | | | | | | |
| **Complications/other treatment** | | | | | | | | | | | | | | | |
| Osteomyelitis/septic arthritis | | | | | | | | | - Yes | | - No | | | | - Not registered |
| Others (describe): | | | | | | | | | | | | | | | |
|  | | | | | | | | | | | | | | | |
| Dead | | - Yes | | - No | | Date: | | | | | | | | | |
| Postmortem exam | | - Yes | | - No | | - Not registered | | | | | | Bacterial findings: | | | |

^1^Blood samples from umbilical cords must not be included

# Supplementary 3. Missing answers in questionnaires

Questionnaires were returned for 171 out of 212 infants with GBS infection (EOBGS = 104/129, LOGBS = 67/83). The percentage of missing answers below is defined as a returned questionnaire with either (1) unanswered question, (2) answer with an “not registered” statement, and (3) implausible or inadequate answers.

| **Clinical variable** | **Missing values (EOD)** | **Missing values (LOD)** | **Missing values (Total)** |
| --- | --- | --- | --- |
| Gestation | 2 (2%) | 3 (5%) | 5 (3%) |
| Birthweight | 3 (3%) | 4 (6%) | 7 (4%) |
| Sex* | 0 (0%) | 0 (0%) | 0 (0%) |
| Region | 3 (3%) | 3 (5%) | 6 (4%) |
| Meningitis* | 0 (0%) | 0 (0%) | 0 (0%) |
| Postnatal age at onset* | -- | -- | 0 (0%) |
| Maternal colonization in vagina or rectum | 57 (55%) | 30 (45%) | 87 (51%) |
| Maternal bacteriuria | 55 (53%) | 29 (43%) | 84 (49%) |
| Maternal fever (>38 °C) | 25 (24%) | 10 (15%) | 35 (20%) |
| Prolonged rupture of membranes (>18h) | 12 (12%) | 8 (12%) | 20 (12%) |
| Previous child with GBS | 22 (21%) | 10 (15%) | 32 (19%) |
| Maternal antibiotics >4h before birth | 15 (14%) | 7 (10%) | 22 (13%) |
| Respiratory symptoms | 8 (8%) | 10 (15%) | 18 (11%) |
| Circulatory symptoms | 10 (10%) | 13 (19%) | 23 (13%) |
| Neurological symptoms | 10 (10%) | 10 (15%) | 20 (12%) |
| Disseminated intravascular coagulation | 12 (12%) | 16 (24%) | 28 (16%) |
| One-minute Apgar score in EOD | 0 (0%) | -- | 0 (0%) |
| Five-minute Apgar score in EOD | 0 (0%) | -- | 0 (0%) |
| Duration of antibiotics to infant | 28 (27%) | 25 (37%) | 53 (31%) |
| Type of antibiotic | 4 (4%) | 6 (9%) | 10 (6%) |
| Presence of osteomyelitis or septic arthritis | 12 (12%) | 13 (19%) | 25 (15%) |
| Mortality | 2 (2%) | 0 (0%) | 2 (1%) |

* Sex, meningitis, and postnatal age at onset were available for all 212 infants

**Supplementary 4. Isolates received from each Danish region**

(A) Number of GBS isolates received from each Danish region during specified time-periods separated by EOD and LOD. Birth year and region was missing for 3 out of 212 total isolates and was therefore not included.

| **Danish Region** | **1999-2001** | **2002-2004** | **2005-2007** | **2008-2009** |
| --- | --- | --- | --- | --- |
| North Denmark Region  EOD  LOD | 3  1 | 4  3 | 1  0 | 1  0 |
| Central Region Denmark  EOD  LOD | 15  10 | 11  11 | 4  9 | 12  0 |
| Region of Southern Denmark  EOD  LOD | 16  4 | 6  5 | 7  7 | 7  4 |
| Region Zealand  EOD  LOD | 1  0 | 1  0 | 0  0 | 1  3 |
| Capital Region of Denmark  EOD  LOD | 2  1 | 6  1 | 0  2 | 3  3 |
| Unknown regions  EOD  LOD | 8  2 | 5  7 | 4  9 | 8  1 |

(B) Number and percentage of serotype and clonal complex combinations received from each Danish region. A total of 33 out of 212 isolates were non-typable or unable to be profiled by multilocus sequence typing and was therefore not included.

| Danish Region | Ia/CC23 | Ib/CC8-10 | III/CC17 | III/CC19 | V/CC1 | Others | Total |
| --- | --- | --- | --- | --- | --- | --- | --- |
| North Denmark Region | 4 (33%) | 1 (8%) | 3 (25%) | 2 (17%) | 1 (8%) | 1 (8%) | 12 (100%) |
| Central Region Denmark | 10 (16%) | 3 (5%) | 25 (40%) | 12 (19%) | 2 (3%) | 11 (17%) | 63 (100%) |
| Region of Southern Denmark | 11 (23%) | 4 (8%) | 17 (35%) | 5 (10%) | 5 (10%) | 6 (13%) | 48 (100%) |
| Region Zealand | 0 (0%) | 0 (0%) | 0 (0%) | 2 (40%) | 1 (20%) | 2 (40%) | 5 (100%) |
| Capital Region of Denmark | 1 (7%) | 3 (21%) | 4 (29%) | 1 (7%) | 1 (7%) | 4 (29%) | 14 (100%) |
| Unknown region | 5 (14%) | 1 (3%) | 24 (65%) | 4 (11%) | 1 (3%) | 2 (5%) | 37 (100%) |

# Supplementary 5. Distribution of serotypes and clonal complexes in EOD and LOD

Frequencies and percentages of serotypes in both EOD and LOD among Danish infants. A total of 5 isolates were non-typable (EOD = 3, LOD = 2) out of 212 total isolates.

|  | **Ia** | **Ib** | **II** | **III** | **IV** | **V** | **IX** | **Total** |
| --- | --- | --- | --- | --- | --- | --- | --- | --- |
| EOD | 32  (25%) | 11  (9%) | 8  (6%) | 57  (45%) | 5  (4%) | 11  (9%) | 3  (2%) | 127  (100%) |
| LOD | 6  (8%) | 4  (5%) | 5  (6%) | 62  (78%) | 1  (1%) | 2  (3%) | 0  (0%) | 80  (100%) |

Frequencies and percentages of clonal complexes in both EOD and LOD among Danish infants. A total of 28 isolates (EOD = 14, LOD = 14) out of 212 total isolates were unable to be profiled by multi locus sequence typing.

|  | **CC1** | **CC8-12** | **CC17** | **CC19** | **CC23** | **Singletons** | **Total** |
| --- | --- | --- | --- | --- | --- | --- | --- |
| EOD | 14  (12%) | 9  (8%) | 36  (31%) | 17  (15%) | 28  (24%) | 11  (10%) | 115  (100%) |
| LOD | 2  (3%) | 5  (7%) | 41  (59%) | 14  (20%) | 7  (10%) | 0  (0%) | 69  (100%) |

#

# Supplementary 6. Distribution of GBS strains by time-period

Percentages of the different GBS strains in specified time-periods in total and separated by EOD and LOD. A total of 33 out of 212 isolates were non-typable or unable to be profiled by multilocus sequence typing and was therefore not included.

# Supplementary 7. GBS strains and other clinical characteristics

Frequencies and percentages of GBS strains within gestational age and one- and five-minute Apgar scores. Data were analyzed by Fisher’s Exact test.

**Premature or term newborns**

|  | **Preterm** | | **Term** | | **Total** | |
| --- | --- | --- | --- | --- | --- | --- |
| **Ia/CC23** | 13 | (52%) | 12 | (48%) | 25 | (100%) |
| **Ib/CC8-10** | 3 | (30%) | 7 | (70%) | 10 | (100%) |
| **III/CC17** | 17 | (32%) | 36 | (68%) | 53 | (100%) |
| **III/CC19** | 10 | (45%) | 12 | (55%) | 22 | (100%) |
| **V/CC1** | 4 | (44%) | 5 | (56%) | 9 | (100%) |
| **Total** | 47 | (40%) | 72 | (60%) | 119 | (100%) |

*p*-value = 0.46

**One-minute Apgar scores in newborn with EOD**

|  | **10-7** | | **6-4** | | **3-0** | | **Total** | |
| --- | --- | --- | --- | --- | --- | --- | --- | --- |
| **Ia/CC23** | 13 | (68%) | 4 | (21%) | 2 | (11%) | 19 | (100%) |
| **Ib/CC8-10** | 5 | (71%) | 0 | (0%) | 2 | (29%) | 7 | (100%) |
| **III/CC17** | 19 | (76%) | 1 | (4%) | 5 | (20%) | 25 | (100%) |
| **III/CC19** | 10 | (77%) | 2 | (15%) | 1 | (8%) | 13 | (100%) |
| **V/CC1** | 6 | (75%) | 1 | (13%) | 1 | (13%) | 8 | (100%) |
| **Total** | 53 | (74%) | 8 | (11%) | 11 | (15%) | 72 | (100%) |

*p*-value = 0.67

**Five-minute Apgar scores in newborn with EOD**

|  | **10-7** | | **6-4** | | **3-0** | | **Total** | |
| --- | --- | --- | --- | --- | --- | --- | --- | --- |
| **Ia/CC23** | 16 | (84%) | 0 | (0%) | 3 | (16%) | 19 | (100%) |
| **Ib/CC8-10** | 6 | (86%) | 0 | (0%) | 1 | (14%) | 7 | (100%) |
| **III/CC17** | 21 | (84%) | 2 | (8%) | 2 | (8%) | 25 | (100%) |
| **III/CC19** | 12 | (92%) | 0 | (0%) | 1 | (8%) | 13 | (100%) |
| **V/CC1** | 7 | (88%) | 0 | (0%) | 1 | (13%) | 8 | (100%) |
| **Total** | 62 | (86%) | 2 | (3%) | 8 | (11%) | 72 | (100%) |

*p*-value = 0.93

# Supplementary 8. GBS strains and treatment duration

The association between GBS strain and the duration of antibiotic treatment in surviving infants. Results are shown as median values with upper interquartile range. Data were analyzed by Kruskal Wallis test with no evidence of differences between strains (*p*-value = 0.53).

# Supplementary 9. Sensitivity analyses

Sensitivity analyses were conducted with recoding of missing answers as the mildest presentation. The associations between GBS strains and disease severity were analyzed by Kruskal Wallis tests, while the association between GBS strains and mortality was analyzed by Fisher’s Exact test.

**Respiratory support**

|  | **None** | | **Mild** | | **Severe** | | **Total** | |
| --- | --- | --- | --- | --- | --- | --- | --- | --- |
| **Ia/CC23** | 10 | (40%) | 10 | (40%) | 5 | (20%) | 25 | (100%) |
| **Ib/CC8-10** | 3 | (27%) | 6 | (55%) | 2 | (18%) | 11 | (100%) |
| **III/CC17** | 22 | (41%) | 19 | (35%) | 13 | (24%) | 54 | (100%) |
| **III/CC19** | 7 | (30%) | 11 | (48%) | 5 | (22%) | 23 | (100%) |
| **V/CC1** | 5 | (50%) | 5 | (50%) | 0 | (0%) | 10 | (100%) |
| **Total** | 47 | (38%) | 51 | (41%) | 25 | (20%) | 123 | (100%) |

No respiratory support (none), nCPAP pressure (mild), or mechanical ventilation (severe)

*p*-value = 0.68

**Circulatory support**

|  | **None** | | **Mild** | | **Severe** | | **Total** | |
| --- | --- | --- | --- | --- | --- | --- | --- | --- |
| **Ia/CC23** | 21 | (84%) | 2 | (8%) | 2 | (8%) | 25 | (100%) |
| **Ib/CC8-10** | 7 | (64%) | 2 | (18%) | 2 | (18%) | 11 | (100%) |
| **III/CC17** | 44 | (81%) | 1 | (2%) | 9 | (17%) | 54 | (100%) |
| **III/CC19** | 15 | (65%) | 5 | (22%) | 3 | (13%) | 23 | (100%) |
| **V/CC1** | 8 | (80%) | 1 | (10%) | 1 | (10%) | 10 | (100%) |
| **Total** | 95 | (77%) | 11 | (9%) | 17 | (14%) | 123 | (100%) |

No circulatory support (none), fluid resuscitation (mild), or vasopressors (severe)

*p*-value = 0.50

**Neurological symptoms**

|  | **None** | | **Mild** | | **Severe** | | **Total** | |
| --- | --- | --- | --- | --- | --- | --- | --- | --- |
| **Ia/CC23** | 15 | (60%) | 8 | (32%) | 2 | (8%) | 25 | (100%) |
| **Ib/CC8-10** | 7 | (64%) | 4 | (36%) | 0 | (0%) | 11 | (100%) |
| **III/CC17** | 23 | (43%) | 21 | (39%) | 10 | (19%) | 54 | (100%) |
| **III/CC19** | 9 | (39%) | 11 | (48%) | 3 | (13%) | 23 | (100%) |
| **V/CC1** | 3 | (30%) | 6 | (60%) | 1 | (10%) | 10 | (100%) |
| **Total** | 57 | (46%) | 50 | (41%) | 16 | (13%) | 123 | (100%) |

No neurological symptoms (none), irritable (mild), or seizures (severe)

*p*-value = 0.24

**Treatment of disseminated intravascular coagulation**

|  | **None** | | **Mild** | | **Severe** | | **Total** | |
| --- | --- | --- | --- | --- | --- | --- | --- | --- |
| **Ia/CC23** | 22 | (88%) | 2 | (8%) | 1 | (4%) | 25 | (100%) |
| **Ib/CC8-10** | 10 | (91%) | 1 | (9%) | 0 | (0%) | 11 | (100%) |
| **III/CC17** | 48 | (89%) | 4 | (7%) | 2 | (4%) | 54 | (100%) |
| **III/CC19** | 20 | (87%) | 3 | (13%) | 0 | (0%) | 23 | (100%) |
| **V/CC1** | 9 | (90%) | 1 | (10%) | 0 | (0%) | 10 | (100%) |
| **Total** | 109 | (89%) | 11 | (9%) | 3 | (2%) | 123 | (100%) |

No treatment of coagulopathy (none), infusion of platelets or fresh frozen plasma (mild), or treatment with AT3 or cryoprecipitate (severe)

*p*-value = 0.99

**Mortality**

|  | **Alive** | | **Dead** | | **Total** | |
| --- | --- | --- | --- | --- | --- | --- |
| **Ia/CC23** | 21 | (84%) | 4 | (16%) | 25 | (100%) |
| **Ib/CC8-10** | 10 | (91%) | 1 | (9%) | 11 | (100%) |
| **III/CC17** | 51 | (94%) | 3 | (6%) | 54 | (100%) |
| **III/CC19** | 18 | (78%) | 5 | (22%) | 23 | (100%) |
| **V/CC1** | 10 | (100%) | 0 | (0%) | 10 | (100%) |
| **Total** | 110 | (89%) | 13 | (11%) | 123 | (100%) |

*p*-value = 0.16
